# Advancing Small-Molecule Immunotherapy Through Polymeric Micelle Delivery

**DOI:** 10.3390/pharmaceutics18040418

**Published:** 2026-03-29

**Authors:** Kiran Suwal, Hyunji Lee, Saroj Bashyal, Donghyun Kim, Hyuk Jun Cho, Duhyeong Hwang

**Affiliations:** 1 College of Pharmacy, Mokpo National University, Muan 58554, Republic of Korea; ksuwal2011@gmail.com; 2 College of Pharmacy, Kyungsung University, Busan 48434, Republic of Korea; hlee@ks.ac.kr; 3 College of Pharmacy, Keimyung University, Daegu 42601, Republic of Korea; sarojbashyal63@gmail.com (S.B.); dkim@kmu.ac.kr (D.K.); hjcho89@kmu.ac.kr (H.J.C.)

**Keywords:** immunomodulators, small-molecule drug, polymeric micelle, drug delivery

## Abstract

Small-molecule immunomodulators have become important components of modern immunotherapy by targeting immune checkpoints, cytokine signaling pathways, metabolic enzymes, and intracellular kinases. Despite pharmacological rationale, many of these agents underperform clinically due to unfavorable physicochemical properties, rapid systemic clearance, limited target accumulation, and dose-limiting toxicities, reflecting inadequate exposure control rather than a lack of target validity. Polymeric micelles, formed through the self-assembly of amphiphilic block copolymers, offer a versatile delivery platform to address these challenges by enhancing solubility, modulating pharmacokinetics, enabling stimuli-responsive release, and facilitating targeted or synchronized co-delivery. In this review, we classify representative small-molecule immunomodulators according to their immunological targets and examine the delivery constraints that shape their therapeutic performance. We then discuss design principles of polymeric micelle systems, including solubilization-driven formulations, microenvironment-responsive architectures, spatial targeting strategies, and co-delivery approaches that align cytotoxic and immunomodulatory mechanisms. Attention is given to the distinction between direct immunomodulators and cytotoxic agents that induce immunogenic cell death, highlighting how micelle-based delivery can enhance efficacy through improved exposure control. By integrating immunopharmacology with formulation science, this review outlines how polymeric micelles may advance the efficacy and safety of small-molecule immunomodulators and identifies key considerations for future translational development.

## 1. Introduction

Small-molecule immunomodulators constitute a mechanistically diverse and increasingly important class of agents in modern immunotherapy [1,2,3]. By targeting immune checkpoints [4,5], cytokine signaling networks [6], metabolic enzymes [7], and intracellular kinases [8], modulate critical regulatory nodes that govern immune activation and suppression across cancer [2], inflammatory [9], and autoimmune diseases [10]. Compared with biologics, small molecules offer practical advantages in synthetic scalability, oral dosing potential, and tissue penetration [11]. Nevertheless, despite compelling mechanistic validation, clinical outcomes frequently fall short of preclinical promise.

Across adaptive, innate, and metabolic immune pathways, therapeutic limitations often arise not from insufficient target validity but from constraints in delivery and exposure control [12]. Lipophilic checkpoint modulators and kinase inhibitors are hindered by poor aqueous solubility and excipient-dependent formulations. Charged innate immune agonists face barriers related to membrane permeability [13,14] and systemic inflammatory toxicity [15]. Metabolic inhibitors, such as those targeting indoleamine 2,3-dioxygenase 1 (IDO1) or adenosine pathways, illustrate that adequate systemic exposure does not necessarily ensure effective pathway suppression within the tumor microenvironment (TME) [16]. In many cases, pharmacokinetics, tissue distribution, and the spatial and temporal regulation of immune signaling exert greater influence on therapeutic outcome than intrinsic molecular potency [17]. These recurring patterns underscore that delivery architecture is a primary determinant of immunological efficacy and safety rather than a secondary formulation concern.

**Figure 1:**
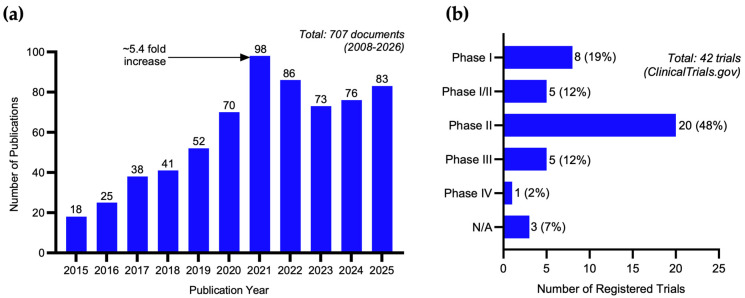
Bibliometric landscape of polymeric micelle-mediated immunotherapy research. (**a**) Annual number of publications retrieved from the Web of Science Core Collection (2015–2025) using the search query: TS = (“polymeric micelle*”) AND (“immunotherapy” OR “immunomodulator*” OR “immune checkpoint” OR “tumor microenvironment” OR “cancer immun*”). A total of 707 documents were identified (2008–2026; accessed March 2026). (**b**) Distribution of registered clinical trials involving polymeric micelle formulations by clinical phase. Data were retrieved from ClinicalTrials.gov using the search terms “polymeric micelle” OR “Genexol” OR “NK105” OR “NC-6004” OR “Nanoplatin” OR “NK012” OR “SP1049C” OR “Nanoxel” (42 studies; accessed March 2026).

The growing recognition of delivery as a critical determinant of immunotherapeutic outcome has catalyzed research activities at the intersection of polymeric nanomedicine and immunopharmacology. A bibliometric analysis of the Web of Science Core Collection using the search terms “polymeric micelle” AND (“immunotherapy” OR “immunomodulator*” OR “immune checkpoint” OR “tumor microenvironment” OR “cancer immun*”) reveals a pronounced upward trend in annual publication output over the past decade, increasing from 18 publications in 2015 to over 83 in 2025 (Figure 1a). This approximately 4.6-fold increase reflects expanding interest in micelle-mediated strategies for immune modulation, paralleling the broader growth of cancer immunotherapy as a clinical paradigm [18]. Notably, research output has accelerated markedly since 2020, coinciding with the clinical maturation of immune checkpoint inhibitors and the increasing emphasis on combination strategies that integrate cytotoxic, metabolic, and immune-modulating agents within nanoscale delivery platforms [19].

The translational relevance of polymeric micelles is further supported by their advancing clinical pipeline. Genexol-PM, a poly(ethylene glycol)–*block*–poly(D,L-lactic acid) (PEG–*b*–PDLLA) micelle formulation of paclitaxel (PTX), has received regulatory approval in South Korea and has been evaluated in multiple phase II and III trials for breast cancer and non-small-cell lung cancer [20,21]. NC-6004, a cisplatin-incorporating PEG–*b*–poly(glutamic acid) micelle, has advanced into phase III evaluations for pancreatic and head-and-neck cancers [22]. NK105, a micellar PTX formulation based on PEG–*b*–poly(aspartate), completed a phase III trial in metastatic breast cancer, although it did not meet its primary endpoint of non-inferiority in progression-free survival [23]. A search of ClinicalTrials.gov using the terms “polymeric micelle” OR “Genexol” OR “NK105” OR “NC-6004” identifies over 42 registered trials as of March 2026, spanning phase I through phase III investigations across multiple solid tumor types (Figure 1b). While most of these trials focus on conventional chemotherapeutic payloads, an emerging subset addresses immunomodulatory combinations and TME-targeting strategies, underscoring the progressive convergence of micellar delivery with immunotherapeutic paradigms [24].

Despite this expanding body of literature, no prior review has systematically analyzed the delivery–biology interface across mechanistically distinct classes of small-molecule immunomodulators within the specific context of polymeric micelle systems. The quantitative growth in this field, together with the clinical precedent established by approved and investigational micelle formulations, underscores the timeliness and necessity of a framework that bridges immunopharmacology with delivery science.

Polymeric micelles, formed through the self-assembly of amphiphilic block copolymers, represent a dynamic and tunable nanoscale delivery platform uniquely positioned to address these challenges [25,26,27]. Unlike rigid nanoparticle systems, micelles exist in thermodynamic equilibrium, allowing modulation of drug–polymer interactions, loading capacity (LC), and release kinetics through rational design of block composition and architecture [28,29]. Their core–shell structure enables solubilization of poorly soluble therapeutic compounds while maintaining colloidal stability in physiological environments [30]. Moreover, micellar systems can be engineered to modulate systemic exposure, respond to tumor-associated physicochemical cues, facilitate tissue- or cell-selective targeting, and synchronize the delivery of multiple agents within defined spatial and temporal windows [31]. Such programmability is relevant for immune-directed therapeutics, where both insufficient activation and uncontrolled systemic stimulation can compromise safety.

Although polymeric micelles have been investigated in oncology drug delivery [32,33,34,35,36], and numerous reviews have addressed the biology of individual immunomodulatory targets, comparatively limited attention has been given to how delivery constraints systematically intersect with mechanistic immunology across small-molecule classes. In particular, the distinction between direct immunomodulators that target immune signaling pathways and cytotoxic agents that induce immunogenic cell death (ICD) highlights the need for delivery systems capable of coordinating both mechanisms under controlled exposure conditions [37,38,39]. A unified analysis of small-molecule immunomodulators through the dual lenses of target biology and delivery architecture remains lacking.

In this review, we organize representative small-molecule immunomodulators according to their immunological targets and examine how physicochemical properties and pharmacokinetic limitations influence therapeutic performance across mechanistic classes. We then analyze core design principles of polymeric micelle systems, including solubilization-driven formulations, stimuli-responsive architectures, spatial targeting strategies, and exposure-synchronizing co-delivery platforms, and evaluate how these approaches may enhance immune modulation while mitigating systemic toxicity. By integrating immunopharmacology with formulation science, this review clarifies the enabling role of polymeric micelles in advancing immune-directed small-molecule therapeutics and outlines key considerations for their rational translational development.

## 2. Small-Molecule Immunomodulators: Target Classes and Delivery Constraints

Small-molecule immunomodulators regulate immune responses by targeting cell-surface receptors, intracellular kinases, or metabolic enzymes that shape immune cell activation and TME dynamics [3,40,41]. While many such agents demonstrate mechanistic rationale and preclinical efficacy, their clinical translation has been constrained by physicochemical limitations, systemic toxicity, or insufficient spatial control within diseased tissues. The following sections summarize representative target classes, emphasizing both their immunological mechanisms and the delivery-related challenges that influence therapeutic outcomes.

### 2.1. Adaptive Immune Regulators

#### 2.1.1. PD-1/PD-L1 Pathway Modulators

Small-molecule inhibitors targeting the programmed cell death protein 1 (PD-1)/programmed death-ligand 1 (PD-L1) axis aim to restore T cell function by disrupting checkpoint-mediated immune suppression [42,43,44]. Engagement of PD-1 with PD-L1 recruits SHP2 phosphatase to the cytoplasmic tail of PD-1, leading to dephosphorylation of key proximal T cell receptor signaling components and attenuation of downstream activation pathways [44,45]. Sustained PD-1 signaling promotes T cell exhaustion, reduced cytokine production, and impaired cytotoxic function within the TME [46]. By interfering with this inhibitory interaction, small-molecule PD-1/PD-L1 modulators seek to reestablish effector T cell activity and enhance antitumor immune responses [47]. Clinical candidates such as CA-170 (Curis/Aurigene), INCB086550 (Incyte), CCX559 (ChemoCentryx/Amgen), IMMH-010 (ImmuneOnco Biopharma), and ABSK043 (Abbisko Therapeutics) have demonstrated the feasibility of checkpoint modulation, employing diverse mechanisms such as PD-L1 dimer stabilization [48], internalization [49], or degradation [50].

Despite these advances, no small-molecule PD-1/PD-L1 inhibitors have yet achieved regulatory approval. Many candidates in this class exhibit limited aqueous solubility, which can complicate formulation and lead to variability in systemic exposure [51]. Thus, formulation optimization and exposure control remain a significant challenge in translating checkpoint modulation into small-molecule format.

#### 2.1.2. RORγt Agonists

The retinoic acid receptor-related orphan receptor gamma t (RORγt) agonists activate RORγt, a lineage-defining transcription factor that regulates Th17 differentiation and effector function [52]. RORγt drives the transcription of IL-17A, IL-17F, and other pro-inflammatory cytokines that contribute to host defense and tissue-specific immune responses [53]. In the TME, controlled Th17 activation has been associated with enhanced recruitment of effector T cells and improved antitumor immunity, although excessive activation may also promote inflammatory pathology or tissue damage [54,55]. Although inhibition of RORγt was pursued in autoimmune disease [53,54,56,57], agonism has emerged as a strategy to boost immune activation in cancer and mucosal immunity [52].

Cintirorgon (LYC-55716) is among the few small-molecule RORγt agonists to advance into oncology trials, where a phase 1 clinical study demonstrated tolerability and pharmacodynamic activity, with a phase 2a trial subsequently announced [58,59]. However, development in this class remains early, and formulation optimization has been limited. Given the hydrophobic nature typical of nuclear receptor ligands, controlled exposure and delivery strategy may be required to balance immune activation with systemic safety.

### 2.2. Innate Immune Activators

#### 2.2.1. STING Agonists

Stimulator of interferon genes (STING) agonists activate innate immunity through the cGAS–STING signaling pathway, which senses cytosolic DNA and triggers downstream activation of TBK1 and IRF3, culminating in robust type I interferon (IFN) production [60]. This cascade promotes dendritic cell maturation, enhances antigen cross-presentation, and facilitates priming of cytotoxic CD8^+^ T cells, thereby bridging innate and adaptive immunity within the TME [61,62]. Sustained STING activation also induces chemokines such as C-X-C motif chemokine ligand (CXCL) 9 and CXCL10, promoting effector T cell recruitment; however, excessive or systemic activation can result in inflammatory toxicity and cytokine-mediated adverse events [63].

Cyclic dinucleotide (CDN) agonists such as ADU-S100 and MK-1454 are highly water soluble and negatively charged, resulting in poor membrane permeability and dependence on specialized delivery systems, often necessitating intratumoral administration to mitigate systemic inflammation [64,65]. Conversely, the small-molecule STING agonist DMXAA (vadimezan) demonstrated species-specific activation of murine but not human STING, underscoring the translational complexity of this target class [66]. Although lipid nanoparticles (LNPs) and polymeric complexes have been widely investigated to enhance the cytosolic delivery of CDNs [67,68,69], polymeric micelle-based strategies remain less established relative to LNP or polyplex approaches. This limitation reflects the intrinsic physicochemical mismatch between charged CDN agonists and the hydrophobic cores of conventional micelles, highlighting a structural boundary of current micelle applicability.

#### 2.2.2. TLR Agonists

Toll-like receptor (TLR) agonists stimulate innate immune signaling pathways, leading to dendritic cell maturation, pro-inflammatory cytokine secretion, and enhanced priming of cytotoxic T lymphocytes [70,71,72]. By amplifying antigen presentation and bridging innate and adaptive immunity, TLR agonists have been explored as cancer immunotherapeutics and vaccine adjuvants [73,74]. However, clinical translation of small-molecule TLR agonists is constrained by delivery-related challenges. Imiquimod, a TLR7/8 agonist approved for topical treatment of actinic keratosis and basal cell carcinoma, suffers from extremely low aqueous solubility and limited tissue penetration, restricting its application primarily to local administration [75]. Resiquimod, another TLR7/8 agonist with broader antitumor potential, exhibits rapid systemic clearance and dose-limiting inflammatory toxicities, complicating systemic delivery [76].

Notably, while various nanocarrier platforms have been investigated to improve the delivery of TLR agonists, polymeric micelle-based formulations remain comparatively less developed and largely at an exploratory stage. These observations position TLR agonists as an emerging, but not yet fully established, class for micelle-enabled immunomodulation. For instance, recent polymeric micelle formulations of resiquimod have demonstrated improved aqueous solubility and attenuated systemic cytokine responses, supporting the feasibility of micelle-based strategies for TLR7/8 agonists [77].

### 2.3. Spatial Tumor Microenvironment Regulators

#### 2.3.1. Chemokine Receptor Antagonists

Chemokine signaling orchestrates immune cell trafficking within tissues through concentration gradients that are essential for immune surveillance, inflammation, and tissue repair [78,79,80]. In cancer, modulation of these pathways is inherently context-dependent, as chemokine axes regulate both immunosuppressive cell recruitment and cytotoxic T cell infiltration, necessitating selective targeting or rational combination strategies to preserve antitumor immunity [80,81]. Blocking pro-tumorigenic axes such as CC motif chemokine ligand (CCL) 2–CCR2 or CCL5–CCR5 can limit the recruitment of immunosuppressive cells and slow tumor progression, whereas inhibiting CXCL9/10–CXCR3 signaling may also hinder effector T cell infiltration [80,82]. Current strategies, therefore, emphasize selective modulation or combination with immune checkpoint inhibitors to optimize therapeutic outcomes.

Maraviroc, a clinically approved CCR5 antagonist for the treatment of HIV-1 infection, demonstrated that chemokine receptor signaling can be safely and effectively modulated in humans [83,84]; however, its low aqueous solubility and moderate oral bioavailability can contribute to variable systemic exposure. Plerixafor (AMD3100), the U.S. Food and Drug Administration (FDA)-approved CXCR4 antagonist used for hematopoietic stem cell mobilization, is highly water soluble but requires parenteral administration, limiting its broader application in chronic immune modulation [85]. Importantly, formulation-driven modulation of pharmacokinetics has been demonstrated for maraviroc using solid drug nanoparticle and nanodispersion approaches, which improved oral absorption and systemic exposure in preclinical models [86,87]. Although these studies did not employ polymeric micelles, they illustrate that physicochemical reformulation alone can substantially reshape the pharmacokinetic and therapeutic behavior of clinically validated chemokine receptor antagonists.

#### 2.3.2. TGF-β Inhibitors

Transforming growth factor-β (TGF-β) signaling establishes an immunosuppressive TME by directly inhibiting effector immune cell function while promoting regulatory and pro-tumorigenic programs [88,89]. Canonical TGF-β signaling is initiated through ligand-induced activation of the type I receptor kinase (activin receptor-like kinase 5; ALK5), which triggers Smad2/3 phosphorylation. The resulting transcriptional programs suppress cytotoxic T cell activity, promote regulatory T cell (Treg) differentiation, impair natural killer cell (NK cell) cytotoxicity, and drive epithelial-to-mesenchymal transition and stromal remodeling [90].

Small-molecule TGF-β inhibitor Galunisertib (LY2157299) provided early clinical proof-of-concept in humans, but also revealed class-wide limitations, including poor aqueous solubility, limited oral bioavailability, and on-target toxicities arising from the pleiotropic roles of TGF-β [91,92]. More recent ALK5 inhibitors, such as vactosertib (TEW-7197), have demonstrated improved antitumor activity and mechanistic synergy with immune checkpoint blockade in preclinical studies [93].

Recent drug delivery studies indicate that spatially confined inhibition of TGF-β signaling can overcome stromal barriers and immune exclusion within tumors [94]. Nanoscale systems incorporating ALK5 inhibitors such as galunisertib or vactosertib have demonstrated improved intratumoral penetration and reduced systemic toxicity in preclinical models [95]. These findings provide a conceptual foundation for extending similar spatial and exposure control strategies to polymeric micelle-based platforms.

#### 2.3.3. SHIP1 Inhibitors

SH2-containing inositol 5′-phosphatase 1 (SHIP1) is an intracellular lipid phosphatase that plays a critical role in immune signal regulation, particularly within hematopoietic lineages [96]. Pharmacological inhibition of SHIP1 has therefore been explored as a strategy to amplify antitumor immunity by relieving Phosphoinositide 3-kinase δ (PI3K)/Akt suppression and enhancing innate immune cell activity [97]. Inhibition of SHIP1 has been shown to promote NK-cell cytotoxicity, expand activating myeloid populations, and shift the tumor immune microenvironment toward a more pro-inflammatory and immune-stimulatory state [98]. These effects position SHIP1 as an emerging immunomodulatory target distinct from classical immune checkpoints that act on T cells.

Several small-molecule SHIP1 inhibitors have been reported, but none have achieved clinical success in oncology [99,100]. The representative steroidal inhibitor 3AC (3α-aminocholestane) demonstrated immune-activating effects in preclinical models; however, its high lipophilicity results in poor aqueous solubility, limited bioavailability, and challenges in exposure control, while prolonged SHIP1 inhibition risks excessive immune activation and hematologic toxicity [101,102]. Similarly, AQX-1125 advanced into clinical testing but was discontinued due to limited efficacy and suboptimal target specificity [103]. These outcomes suggest that the therapeutic potential of SHIP1 inhibition depends not only on target biology but also on delivery strategies capable of achieving controlled and spatially restricted modulation.

### 2.4. Metabolic Immune Suppression Targets

#### 2.4.1. IDO1 Inhibitors

IDO1 is overexpressed in tumor and antigen-presenting cells, where it catalyzes tryptophan (Trp) degradation into kynurenine (Kyn) and promotes immune evasion within the TME [104]. Trp depletion activates the general control nonderepressible 2 (GCN2) stress-response pathway in effector T cells, leading to cell cycle arrest and functional anergy. Concurrently, accumulated Kyn engages aryl hydrocarbon receptor (AhR) signaling to drive Treg differentiation and reinforce T cell exhaustion [105,106]. In parallel, sustained IDO1 activity induces a tolerogenic phenotype in antigen-presenting cells, further amplifying local immune suppression. IDO1 inhibitors seek to restore antitumor immunity by blocking Trp catabolism and reducing Kyn production [106]. This dual metabolic intervention alleviates GCN2-mediated amino acid stress and dampens Kyn–AhR signaling, thereby promoting effector T cell recovery and limiting Treg expansion [107]. Such multifaceted immune reprogramming provides the rationale for combining IDO1 inhibitors with immune checkpoint blockade or chemotherapy [108].

Despite strong mechanistic rationale and encouraging preclinical results, clinical translation of IDO1 inhibitors has been hindered by pronounced delivery-related challenges. Representative compounds such as NLG919 and indoximod exhibit poor aqueous solubility, short systemic half-life (t_½_), and limited tumor accumulation, and mismatched pharmacokinetic profiles further complicate combination regimens [109,110]. Consequently, adequate systemic dosing does not reliably translate into effective local suppression of the Kyn pathway, positioning IDO1 inhibitors as a paradigm in which delivery, rather than target potency, dictates therapeutic outcome.

#### 2.4.2. Arginase Inhibitors

Arginase catalyzes the hydrolysis of L-arginine to urea and ornithine, depleting L-arginine, which is essential for T cell proliferation and function [111]. As a result, increased arginase activity leads to immune suppression, promoting tumor growth and allowing cancer cells to evade immune surveillance. Arginase inhibitors are a class of therapeutic agents that block the activity of arginase enzymes, particularly arginase I, which is expressed in myeloid-derived suppressor cells and tumor-associated macrophages within the TME.

Arginase inhibitors restore L-arginine levels in the TME, counteracting metabolic immune suppression [112]. CB-1158 has progressed into clinical evaluation, mainly in combination with immune checkpoint inhibitors, but has shown limited single-agent efficacy [113]. OAT-1746 has demonstrated similar preclinical promise [114]. These findings suggest that effective arginase-targeted immunotherapy may require combination regimens and delivery strategies that ensure sufficient tumor-restricted exposure while minimizing systemic metabolic perturbation.

#### 2.4.3. A2A Adenosine Receptor and CD39/CD73 Antagonists

The adenosine signaling pathway is a major immunosuppressive mechanism within the TME [115]. Under hypoxic or inflammatory conditions, extracellular adenosine triphosphate (ATP) is sequentially converted to adenosine by CD39 and CD73, leading to suppression of T cell and NK cell function through activation of A2A adenosine receptors (A2AR) [116]. Accordingly, A2AR antagonists and CD39/CD73 inhibitors have been explored to restore antitumor immunity.

Several small-molecule A2AR antagonists have advanced into clinical evaluation. CPI-444 (ciforadenant) is an orally bioavailable A2AR antagonist that has shown early clinical activity, particularly in combination with immune checkpoint inhibitors [117]. AZD4635 is another orally administered A2AR antagonist under investigation in multiple solid tumors [118]. Despite favorable drug-like properties and manageable systemic exposure, both agents have demonstrated limited efficacy as monotherapies, underscoring the complexity and redundancy of immunosuppressive pathways in tumors.

From a delivery perspective, these outcomes highlight a key limitation of A2AR antagonists: adequate systemic exposure does not guarantee effective intratumoral pathway inhibition, especially in immunologically “cold” tumors with restricted immune infiltration. This disconnect positions the adenosine pathway as a class where delivery-mediated spatial control, rather than further optimization of oral bioavailability, may be required to achieve robust immunomodulatory effects.

#### 2.4.4. AhR Antagonists

AhR is a ligand-activated transcription factor that plays an important role in immune regulation and tumor-associated immune suppression [119,120]. In the TME, AhR is frequently activated by endogenous ligands such as Kyn, a metabolite of Trp produced by IDO1 and TDO2, leading to transcriptional programs that promote Treg differentiation and suppress antitumor immune responses [121]. Accordingly, AhR antagonists are being explored as cancer immunotherapy agents to reverse immune suppression.

Several small-molecule AhR antagonists have entered early clinical evaluation, including BAY 2416964 and IK-175, the latter being investigated in combination with immune checkpoint inhibitors [122,123]. However, AhR signaling also plays pleiotropic roles in normal physiology, including maintenance of barrier function, microbiome homeostasis, and tissue integrity in organs such as the gut and lungs [124]. From a delivery perspective, these considerations highlight the potential need for spatially confined or tumor-restricted AhR inhibition to balance immunotherapeutic efficacy with systemic safety.

### 2.5. Immune-Modulating Kinase Targets

#### 2.5.1. PI3K-δ Inhibitors

PI3K-δ is predominantly expressed in leukocytes and functions as a key regulator of PI3K/Akt signaling downstream of the B-cell receptor, cytokine receptors, and costimulatory pathways [125,126,127]. PI3K-δ inhibitors are small-molecule kinase inhibitors with both direct antitumor and immunomodulatory effects, particularly in B-cell malignancies such as chronic lymphocytic leukemia and follicular lymphoma [128]. By inhibiting PI3K/Akt signaling downstream of the B-cell receptor and cytokine pathways, these agents suppress malignant B-cell proliferation while modulating immune cell function within the TME.

Despite strong target engagement, clinical experience with first-generation inhibitors such as idelalisib and duvelisib has revealed limitations driven by systemic exposure rather than insufficient efficacy [129,130,131]. Poor aqueous solubility, narrow therapeutic windows, and immune-related toxicities—including hepatotoxicity, colitis, and infections—have constrained long-term use. Although next-generation inhibitors such as umbralisib and zandelisib were developed to improve selectivity and tolerability, delivery, and exposure control challenges remain central to optimizing therapeutic outcomes in this class [128]. Notably, polymeric micelle-based formulations of idelalisib have been reported to improve aqueous solubility and modulate systemic exposure in preclinical models. Although still at an early stage, such studies suggest that micellar delivery may help mitigate exposure-related toxicities while preserving antitumor efficacy.

#### 2.5.2. BTK Modulators

Bruton’s tyrosine kinase (BTK) is a central mediator of B-cell receptor signaling and innate immune activation, playing a critical role in B-cell maturation, survival, and cytokine production [132]. Dysregulated BTK activity is a hallmark of many B-cell malignancies, and pharmacological BTK modulation has demonstrated substantial clinical benefit in diseases such as chronic lymphocytic leukemia, mantle cell lymphoma, and Waldenström’s macroglobulinemia [133].

First-generation covalent BTK inhibitors, exemplified by ibrutinib, irreversibly inhibit BTK by targeting the Cys481 residue and effectively suppress malignant B-cell proliferation [134,135]. However, clinical experience revealed that long-term efficacy is often constrained by systemic exposure and off-target immune perturbation rather than insufficient BTK inhibition. Subsequent generations of inhibitors, including acalabrutinib and zanubrutinib, were developed to improve selectivity and reduce adverse events, while non-covalent agents such as pirtobrutinib address resistance driven by BTK C481 mutations [136,137,138]. In parallel, BTK degraders based on proteolysis-targeting chimeras have emerged as an alternative strategy capable of eliminating BTK protein and overcoming both kinase-dependent and kinase-independent resistance [139,140].

From a delivery perspective, BTK modulators exemplify immunomodulatory agents for which therapeutic outcome is strongly influenced by exposure control and systemic immune balance. These characteristics suggest that strategies enabling controlled exposure, tissue-restricted modulation, or temporal limitation of BTK inhibition may enhance therapeutic benefit while minimizing systemic immune disruption.

## 3. Polymeric Micelles as Delivery Platforms for Immunomodulatory Small Molecules

Small-molecule immunomodulators frequently underperform in vivo not solely due to insufficient target engagement, but because systemic administration does not reliably translate into spatially confined immune modulation within diseased tissues [1,3]. Polymeric micelles offer a versatile delivery platform to address these constraints by enabling solubilization [141], controlled release [142], spatial targeting [19], and synchronized co-delivery of immunomodulatory agents [143]. Rather than organizing micelle systems by therapeutic indication, this section summarizes recent advances according to core design principles that govern delivery behavior and immune response modulation. A comprehensive overview of representative micelle-based formulations across different immunomodulator classes is provided in Table 1.

Although not all the agents discussed are classical immune-targeted modulators, several cytotoxic or immunosuppressive small molecules exert indirect or context-dependent effects on immune function. Certain cytotoxic agents induce ICD, characterized by calreticulin exposure on the tumor cell surface, extracellular ATP release, and high mobility group box 1 (HMGB1) secretion [152,153,154,155]. These danger-associated molecular patterns promote dendritic cell recruitment and maturation, enhance antigen cross-presentation, and ultimately support cytotoxic CD8^+^ T cell priming. Polymeric micelles can amplify these ICD-driven immune responses by improving intracellular delivery and spatial control of drug exposure within the TME.

### 3.1. Solubilization-Driven Micelles: Enabling Systemic Administration of Poorly Soluble Immunomodulators

Solubilization constitutes the most fundamental and often enabling function of polymeric micelles in the delivery of poorly soluble small-molecule immunomodulators [26,156]. Amphiphilic block copolymers such as PEG–*b*–polyester systems (e.g., PEG–*b*–poly(lactic-co-glycolic acid) (PEG–*b*–PLGA), PEG–*b*–poly(ε-caprolactone) (PEG–*b*–PCL), poly(2-oxazoline)-based triblock copolymers (POx), and PEG–*b*–poly(lactic acid) derivatives self-assemble above the critical micelle concentration (CMC) into dynamic core–shell nanostructures. The hydrophobic core provides a microenvironment that stabilizes poorly soluble molecules through hydrophobic matching [157], van der Waals interactions [158], and in certain systems, π–π stacking [159,160] or hydrogen bonding [161]. Meanwhile, the hydrophilic corona ensures colloidal stability and reduced protein adsorption in biological media [162]. This equilibrium-based architecture can increase apparent aqueous solubility by orders of magnitude and mitigate reliance on surfactant-rich formulations that contribute to systemic toxicity [163].

**Figure 2:**
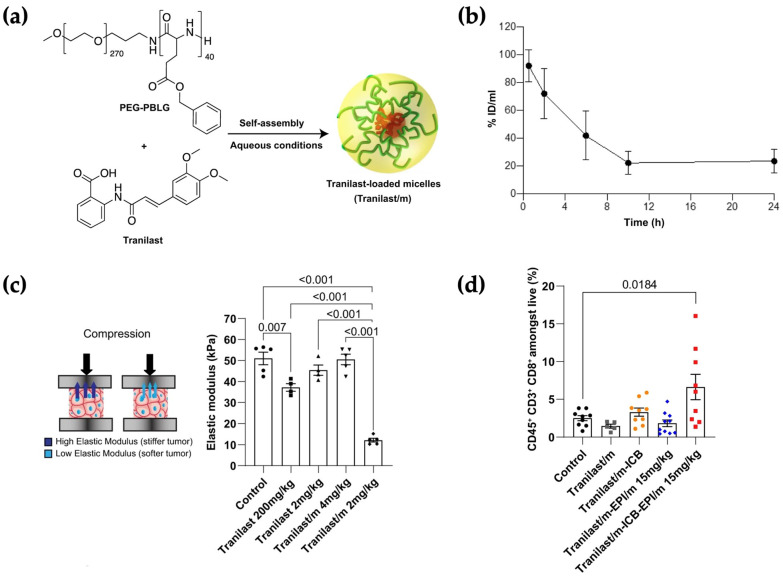
Tranilast-loaded PEG–*b*–PBLG micelles prolong systemic exposure and remodel the TME to enhance antitumor immunity. (**a**) Schematic of tranilast-loaded micelles (tranilast/m) preparation. The micelles were self-assembled by mixing PEG–*b*–PBLG and tranilast in aqueous conditions. (**b**) Time-dependent decay of plasma concentration after intravenous injection of 2 mg/kg Tranilast/m on a tranilast basis. Data shown as the mean ± SD (*n* = 4 mice). (**c**) Ex vivo elastic modulus quantification of E0771 tumor specimens (3 × 2 × 2 mm, from the tumor interior) following unconfined compression to a final strain of 30% with a strain rate of 0.1 mm/min (*n* = 4–5 mice). (**d**) Percentages of intratumoral CD45^+^ CD3^+^ CD8^+^ (SP, single positive) cell population gated on live cells in different treatment groups, measured by flow cytometry (*n* = 5–9 mice). Data are presented as mean ± SE, statistical analyses were performed by comparing means between two independent groups using the unpaired parametric Welch *t* test. (Adapted from [144], CC BY 4.0.).

A representative example of solubilization-enabled immunomodulation is provided by the micellar formulation of tranilast, a hydrophobic anti-fibrotic agent repurposed for TME modulation. Panagi et al. formulated tranilast using PEG–*b*–poly(benzyl-L-glutamate) (PBLG) to generate micelles with an average diameter of ~90–95 nm, low polydispersity index (PDI ≈ 0.12), and drug LC of approximately 10 wt% (Figure 2a). Micellar encapsulation markedly altered systemic pharmacokinetics, extending plasma t_½_ (terminal t_½_ ≈ 21 h) compared with the rapid clearance of free tranilast (≈98% eliminated within 2 h) (Figure 2b) [144]. Beyond improved systemic stability, micellar tranilast enhanced cancer-associated fibroblast uptake, more effectively suppressed TGF-β signaling, reduced tumor stiffness and interstitial fluid pressure, and improved vascular perfusion, thereby facilitating intratumoral accumulation of co-administered nanomedicines (Figure 2c). Importantly, these microenvironmental changes translated into enhanced CD8^+^ T cell infiltration and improved response to immune checkpoint blockade (Figure 2d). This study illustrates how solubilization-driven micelle design can extend beyond formulation feasibility to reprogram tumor mechanics and immune accessibility, ultimately amplifying downstream immunotherapeutic efficacy.

The impact of solubilization is particularly evident for poorly soluble immunomodulators such as the PI3K-δ inhibitor idelalisib and the TLR7/8 agonist resiquimod [145]. In the POx micelle system, each compound was first formulated as a single-drug micelle with high incorporation efficiency and nanoscale size. Resiquimod and idelalisib were loaded with high loading efficiencies (LE) (92.4 wt% and 99.3 wt%, respectively) and high LCs (42.4 wt% for resiquimod and 44.2 wt% for idelalisib), yielding monodisperse nanoparticles with diameters of 27.6 nm (PDI 0.21) and 33.8 nm (PDI 0.14), respectively. Idelalisib micelles remained colloidally stable for up to 18 days at 4 °C, whereas resiquimod single-drug micelles exhibited early instability, underscoring the importance of drug–polymer compatibility. Although single-drug micelles primarily improved systemic handling and exposure feasibility rather than producing dramatic therapeutic benefit alone, these data demonstrate that high-capacity micellar solubilization enables clinically relevant dosing of otherwise formulation-limited immunomodulators, establishing the foundation for subsequent exposure-synchronized combination strategies.

Collectively, these findings indicate that solubilization is not merely a formulation convenience but a prerequisite step that determines whether mechanistically validated immunomodulators can achieve therapeutically meaningful exposure within the TME.

### 3.2. Stimuli-Responsive Micelles: Tumor Microenvironment-Triggered Release and Immune Reprogramming

Beyond solubilization, polymeric micelles can be rationally engineered to respond to tumor-associated physicochemical cues such as acidic pH, elevated glutathione (GSH) levels, reactive oxygen species (ROS), or disease-specific enzymatic activity [164]. These stimuli-responsive systems enable spatially and temporally controlled drug release, thereby reducing off-target exposure while amplifying local immune modulation [165,166]. Importantly, such designs allow synchronization of chemotherapy-induced ICD with direct immunomodulatory signaling, integrating cytotoxic and immune-directed mechanisms within a single platform.

#### 3.2.1. pH-Responsive Micelles: Exploiting Acidic Tumor and Lysosomal Microenvironments

The mildly acidic extracellular TME (pH~6.5–6.8) and the more pronounced acidity of endolysosomal compartments (pH~5.0–6.0) provide spatially restricted biochemical cues that can be harnessed to trigger micellar disassembly [167,168,169,170,171,172,173,174]. Polymers incorporating protonatable tertiary amines, imidazole groups, or acid-labile linkages remain relatively hydrophobic and structurally stable at physiological pH (7.4), thereby preserving systemic integrity and minimizing premature drug leakage [169,175]. Upon exposure to acidic environments, protonation increases polymer hydrophilicity, electrostatic repulsion, and osmotic swelling, destabilizing the hydrophobic core and accelerating intracellular drug release [175,176,177].

In immunotherapy settings, this endolysosome-coupled release is particularly advantageous because many immunomodulators—including TLR agonists and certain chemotherapeutics that induce ICD—require intracellular or endosomal engagement to activate immune signaling pathways [146,178,179]. By restricting payload liberation to acidic tumor compartments, pH-responsive micelles enhance on-target immune activation while mitigating systemic inflammatory toxicity.

**Figure 3:**
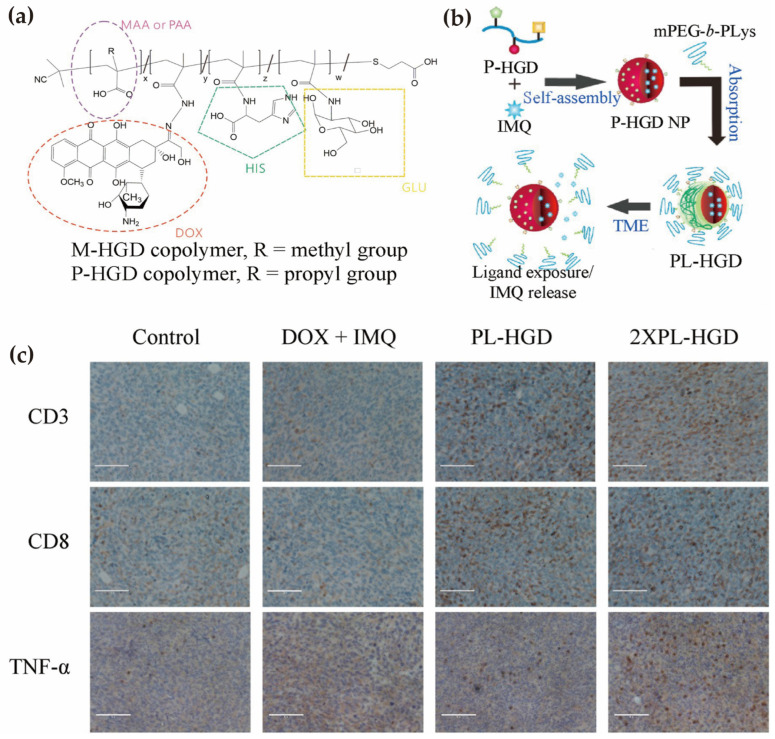
pH-triggered micellar activation and immune amplification in the TME. (**a**) The structure of M-HGD and P-HGD copolymers. (**b**) IMQ-containing P-HGD nanoparticles were prepared using a dialysis method. Methoxy poly(ethylene glycol) (mPEG)–*b*–PLys copolymers were then attached onto P-HGD nanoparticles through electrostatic interactions to form PL-HGD micelles. (**c**) Immunohistochemistry images of tumor sections stained with CD3, CD8, and TNF-α antibodies. The scale bar is 100 μm. (Adapted from [146], licensed under CC BY 4.0.). Scale bar: 100 μm.

A representative example is the dual pH-sensitive micellar system developed by Wen et al., in which poly(propyl methacrylate-co-glucosamine/histidine/doxorubicin (DOX))-based nanoparticles were shielded with methoxy PEG–*b*–P(L-lysine) to maintain stability at physiological pH (7.4) (Figure 3a) [146]. Under mildly acidic TME conditions, protonation of propylacrylic acid and histidine residues induced PEG deshielding and exposed glucose ligands, enhancing tumor uptake and enabling partial extracellular release of the TLR7 agonist imiquimod (Figure 3b). Subsequent endolysosomal acidification triggered hydrazone bond cleavage and intracellular DOX release. Functionally, this spatially staged release synchronized DOX-induced ICD with imiquimod-mediated dendritic cell activation and M1 macrophage polarization, resulting in enhanced CD8^+^ T cell infiltration and significant tumor growth inhibition compared with free-drug combinations (Figure 3c).

Complementary design principles were demonstrated by Yan et al., who utilized poly((1,4-butanediol) diacrylate-β-5-hydroxyamylamine)(PDHA)–*b*–PEG micelles to couple irinotecan-induced ICD with TLR7/8 activation through lysosome-triggered protonation [180]. Similarly, Ma et al. engineered a poly(oligo(ethylene glycol) methyl ether methacrylate)–*b*–poly(2-(diisopropylamino)ethyl methacrylate) block copolymer with a tuned pKa (~6.3–6.4), enabling tumor-acid-triggered micelle destabilization and synergistic co-delivery of a STING agonist and a chemotherapeutic agent [181]. These systems collectively illustrate that rational pKa engineering can translate tumor acidity into coordinated chemo-immunotherapeutic amplification.

Building upon such chemically responsive frameworks, more advanced pH-sensitive platforms integrate additional targeting and immune-modulatory functions. For example, Du et al. incorporated tumor-associated macrophage membrane cloaking into hyaluronic acid-*grafted*-poly(histidine) micelles, coupling imidazole protonation-driven swelling with the colony stimulating factor 1 scavenging to reduce M2 macrophage prevalence and enhance CD8^+^ T cell infiltration [182]. Together, these studies demonstrate that pH-responsiveness can extend beyond simple release control to orchestrate multi-level immune reprogramming within the TME.

#### 3.2.2. Redox-Responsive Micelles: Leveraging Elevated Glutathione Levels

Tumor cells frequently exhibit elevated intracellular GSH concentrations (millimolar range) compared with extracellular fluids, creating a reductive gradient that can be exploited for selective intracellular drug release [183,184]. Redox-responsive polymeric micelles typically incorporate disulfide (–S–S–) linkages within the polymer backbone or as core crosslinkers, which remain stable in circulation but undergo rapid cleavage in high-GSH environments following cellular internalization [185,186,187,188]. This reduction-triggered destabilization enables spatially confined cytosolic drug liberation while minimizing premature systemic release.

Chiang et al. developed a GSH-responsive amphiphilic polymer containing reducible disulfide bonds within the micellar core to enable intracellularly triggered release [189]. In vitro release profiles demonstrated markedly accelerated drug liberation in high-GSH conditions relative to physiological buffer, confirming reduction-dependent micelle destabilization. In vivo, the redox-responsive formulation improved tumor growth inhibition compared with free drug and was associated with enhanced CD8^+^ T cell accumulation and modulation of immune-related signaling pathways within the TME.

Redox-responsive crosslinking has also been integrated with active targeting strategies. Xiao et al. designed LHRH-targeted, disulfide-crosslinked micelles for triple-negative breast cancer (TNBC), in which reductive cleavage simultaneously destabilized the core and released PTX intracellularly [190]. This strategy improved tumor selectivity, enhanced cytotoxic efficacy, and strengthened downstream immune activation compared with non-responsive controls. Collectively, these systems demonstrate that GSH-sensitive micelles translate intracellular reductive gradients into controlled exposure and amplified immunotherapeutic outcomes.

#### 3.2.3. ROS- and Mitochondria-Responsive Systems: Amplifying Immunogenic Stress

Beyond reductive responsiveness, ROS-responsive micelles exploit the intrinsically oxidative TME to amplify immunogenic stress [191,192]. Unlike simple disulfide-cleavage systems, ROS-responsive architectures often incorporate oxidation-labile linkages (e.g., thioether groups) or photosensitizers that convert oxidative signals into enhanced cytotoxicity and immune activation. In these systems, ROS functions not only as a release trigger but also as a biological amplifier of ICD.

Hu et al. developed a ROS-responsive amphiphilic block copolymer (pep-PAP) incorporating thioether-containing segments that undergo oxidation under elevated ROS conditions [193]. Upon oxidative conversion of thioether to sulfoxide/sulfone, micellar core hydrophobicity decreased, leading to destabilization and accelerated PTX release. The micelles were additionally functionalized with an anti-PD-L1 peptide to promote multivalent PD-L1 binding and lysosomal degradation. In TNBC models, ROS-triggered release enhanced intratumoral PTX accumulation, reversed PTX-induced PD-L1 upregulation, increased CD8^+^ T cell infiltration, elevated IFN-γ and IL-2 levels, and achieved substantial tumor growth inhibition (~78%), illustrating how ROS-responsive design can couple chemotherapy-induced ICD with checkpoint modulation.

**Figure 4:**
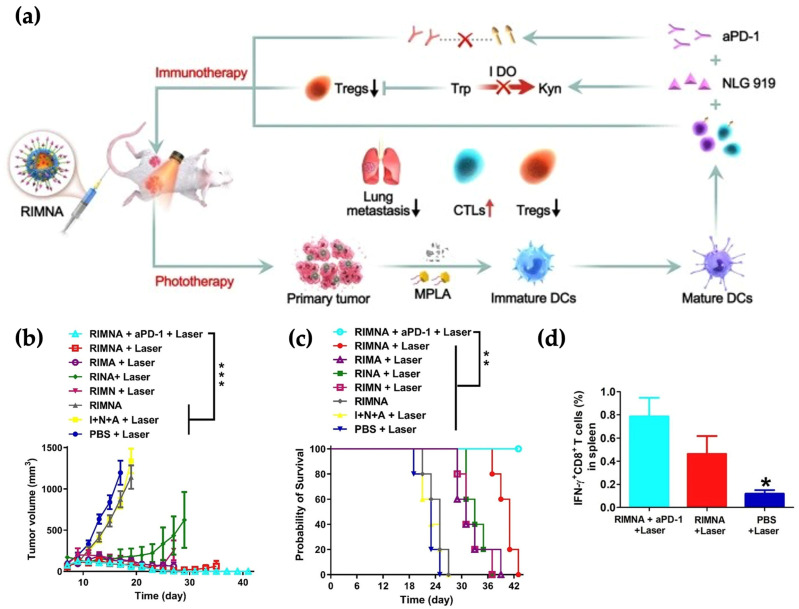
ROS-amplified photoimmunotherapy with IDO inhibition synergizes with PD-1 blockade to induce complete tumor regression. (**a**) Schematic illustration of RIMNA polymeric micelles as an amplifier for robust photoimmunotherapy synergized with PD-1 blockade antibody against primary tumors, distant tumors, and lung metastasis. (**b**) Volume change in the primary tumor. (**c**) Corresponding survival curves. (**d**) Anti-metastasis effect on IFN-γ + CD8^+^ T cells. Data were displayed as mean ± SEM (*n* = 5, * *p* < 0.05, ** *p* < 0.01, *** *p* < 0.001 vs. RIMNA + aPD-1 + Laser group). Arrows indicate the direction of change: ↓ indicates decrease, and ↑ indicates increase. (Reprinted from [148], licensed under CC BY 4.0.).

Huang et al. further demonstrated that ROS amplification can synergize with metabolic and innate immune modulation, as shown in Figure 4 [148]. Their system integrated an IDO inhibitor, MPLA, and phototherapeutic agents within a redox/ROS-sensitive micellar platform. Near-infrared irradiation enhanced ROS generation, which intensified ICD hallmarks, including calreticulin exposure and ATP secretion. Combination with PD-1 blockade produced complete tumor regression in murine colon cancer models, underscoring that ROS-triggered stress can potentiate adaptive immune responses.

Mitochondria-targeted ROS strategies add an additional layer of spatial precision [194]. By directing photosensitizers or ROS-generating components to mitochondria, oxidative stress can be concentrated within this organelle, intensifying ICD signaling and dendritic cell activation. Feng et al. and related studies showed that mitochondrial ROS amplification increased HMGB1 release, enhanced antigen presentation, and promoted durable cytotoxic T cell responses [195].

Finally, Peng et al. demonstrated that photothermal therapy (PTT)-associated oxidative stress can paradoxically induce adaptive immune resistance (e.g., PD-L1 and IDO upregulation) in residual tumor margins [196]. Micellar co-delivery of a photosensitizer with the IDO inhibitor NLG919 mitigated this resistance, suppressed marginal tumor recurrence, and promoted systemic antitumor immunity (abscopal effect). These findings emphasize that ROS-responsive micelles can convert therapy-induced oxidative stress from a pro-tumor adaptation into a sustained immunogenic signal.

Together, ROS- and mitochondria-responsive micelles function as immune amplifiers rather than passive carriers. By integrating oxidative stress generation, controlled release, and checkpoint or metabolic modulation, these platforms translate tumor redox imbalance into coordinated chemo-immunotherapeutic reprogramming.

#### 3.2.4. Enzyme-Responsive Micelles: Exploiting Disease-Specific Metabolic Signatures

Enzyme-responsive micelles leverage tumor-overexpressed enzymes to induce site-specific structural destabilization and drug release. Unlike pH- or redox-sensitive systems that rely on physicochemical gradients, these platforms incorporate enzyme-cleavable peptide linkers or enzyme-reactive polymer segments that undergo selective degradation within the TME, thereby coupling biochemical activity to spatially restricted exposure control.

Protease-triggered micellar systems have been explored to enhance intracellular drug liberation. For example, Wan et al. developed cathepsin B-responsive micelles incorporating a GFLG peptide sequence, which was cleaved in enzyme-rich tumor tissues to promote accelerated DOX release and improved tumor growth inhibition [197]. Although primarily designed to enhance chemotherapeutic efficacy, this study highlights how protease-sensitive structural elements can translate tumor-associated enzymatic activity into controlled payload release.

A mechanistically distinct and immunologically integrated approach was reported by Park et al., who constructed PEG–*b*–poly(Trp)-based micelles in which the hydrophobic core itself functioned as a substrate for IDO [149]. In IDO-overexpressing tumors, enzymatic oxidation of Trp residues into Kyn increased core hydrophilicity, induced micellar swelling, and triggered localized drug release. When loaded with the IDO inhibitor NLG919, the system not only enhanced intratumoral drug exposure but also suppressed Kyn-mediated immunosuppression. In vivo, IDO-responsive micelles reduced the [Kyn]/[Trp] ratio, increased CD8^+^ T cell infiltration, and decreased Treg populations, demonstrating that metabolic enzyme-triggered carrier destabilization can be directly coupled to immune pathway modulation. By converting tumor-specific enzymatic activity into both controlled drug delivery and immunological reprogramming, this platform exemplifies how enzyme-responsive polymers can function as active regulators of the tumor immune microenvironment rather than passive carriers.

### 3.3. Spatially Targeted Micelles: Restricting Immune Modulation to Specific Cells and Tissues

Precise spatial control is particularly important for immunomodulators whose systemic activation can lead to toxicity or immune dysregulation. Polymeric micelles can achieve spatial targeting through passive accumulation in leaky vasculature or through active targeting via surface-displayed ligands that recognize receptors on tumor cells or immune cell subsets.

Xinyun Qiu et al. developed ATN-MPTX micelles—α5β1 integrin-targeted and reduction-sensitive polymeric micelles—for TNBC [150]. Due to its immunosuppressive TME and the absence of effective targeted therapies, TNBC has a poor prognosis. To address this, micelles were developed for use in chemo-immunotherapy. The ATN-MPTX micelles demonstrated high drug loading (up to 23.1 wt%), excellent tumor selectivity, and enhanced stability due to disulfide crosslinking. Compared to free PTX and non-targeted micelles, ATN-MPTX induced stronger ICD, marked by higher calreticulin exposure, ATP, and HMGB1 release. These effects led to enhanced dendritic cell maturation, macrophage polarization to the M1 phenotype, and increased infiltration of CD8^+^ and CD4^+^ T cells, while reducing Tregs. When combined with a nano-formulated STING agonist (CPs-CDN), ATN-MPTX showed synergistic chemo-immunotherapy, significantly suppressing primary tumor growth and lung metastasis in TNBC mouse models, while maintaining a favorable safety profile. The study highlights ATN-MPTX as a potent platform for targeted chemo-immunotherapy, capable of converting “cold” tumors into “hot,” immunoresponsive ones.

Examples include integrin-targeted micelles designed for TNBC, which enhance tumor accumulation and induce robust ICD while minimizing systemic exposure. Other systems employ immune cell membrane cloaking or receptor-specific peptides to direct immunomodulators toward tumor-associated macrophages or dendritic cells, thereby reshaping the local immune landscape without triggering widespread inflammation.

Although this review focuses on polymeric micelles, an instructive exception is provided by PLGA-based polymeric nanoparticles that apply the same “spatial immune programming” logic. Kim et al. encapsulated two newly developed TLR7/8 bi-specific imidazoquinoline-based ester agonists (the racemic mixture ‘522’ and its S-configured stereoisomer ‘528’) into PLGA nanoparticles to enhance delivery to antigen-presenting cells [198]. Rather than addressing solubility alone, PLGA encapsulation increased dendritic cell activation and improved antigen presentation via MHC I compared with soluble agonists. Following subcutaneous administration, the nanoparticles migrated to draining lymph nodes, where they promoted localized dendritic cell activation/expansion and enabled stronger antigen-specific CD8^+^ T cell priming while limiting systemic inflammatory exposure. This example illustrates how polymeric nanoformulations—even when not micelles—can enforce anatomical restriction of innate immune activation to lymphoid compartments, thereby improving the balance between immune potency and safety.

Such spatially targeted micelles illustrate how delivery design can decouple immune activation from systemic toxicity, a recurring limitation of many small-molecule immunomodulators.

### 3.4. Co-Delivery and Exposure Synchronization: Micelles as Platforms for Chemo-Immunotherapy Integration

Combination therapy is central to modern cancer immunotherapy, yet co-administration of agents with distinct physicochemical properties and pharmacodynamic profiles frequently results in asynchronous exposure within tumors [199,200,201]. Polymeric micelles offer a unique solution by enabling co-encapsulation and spatially coordinated release of multiple therapeutics within a single nanoscale assembly. Beyond simple drug combinations, micellar systems can be rationally engineered to synchronize cytotoxic stress, checkpoint modulation, metabolic inhibition, or cytokine signaling within defined tumor compartments, thereby converting parallel therapies into mechanistically aligned immunological amplification.

Nanomicellar PTX preserves PTX-induced ICD—including calreticulin exposure, ATP release, and HMGB1 secretion—while reducing nonspecific cytotoxicity toward immune cells. This enables low-dose PTX to function as an in situ vaccine-like trigger that enhances dendritic cell maturation and intratumoral CD8^+^ T cell infiltration [202]. However, nano-PTX monotherapy can induce adaptive immune resistance, characterized by PD-1 upregulation on CD8^+^ T cells and increased PD-L1 expression within the TME, indicating that cytotoxic ICD must be temporally aligned with checkpoint blockade to achieve sustained immune amplification.

**Figure 5:**
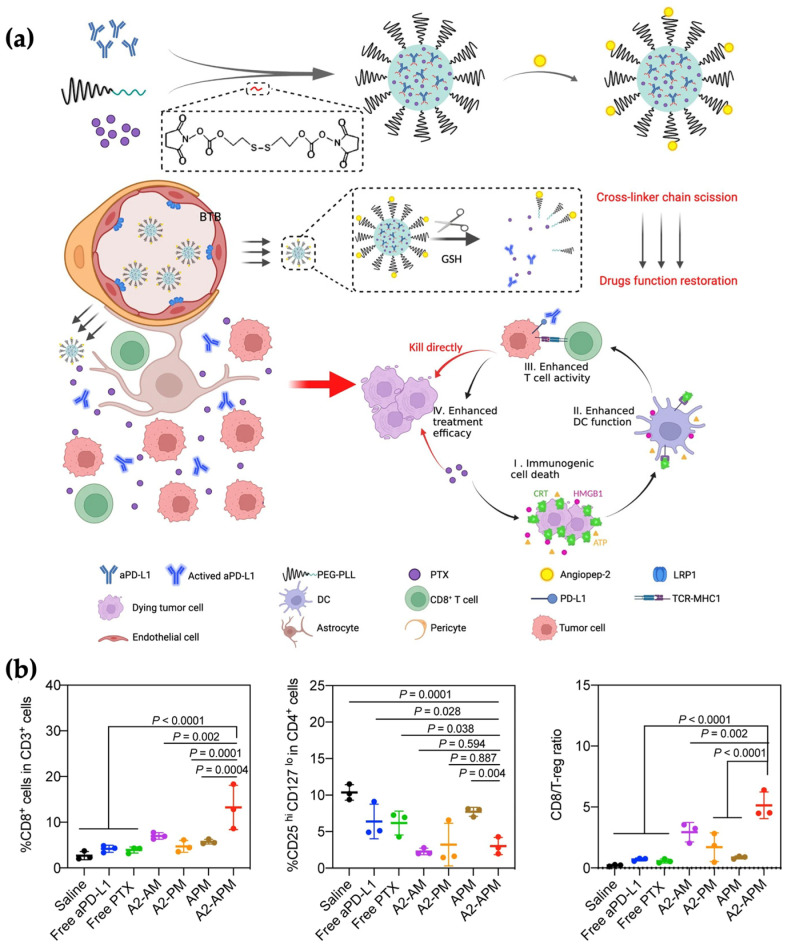
(**a**) Schematic illustration of A2-APM structure and strategy for enhanced ICB therapy against GBM. A redox-responsive micelle was developed, which covalently linked massive aPD-L1 via recoverable crosslinkers. PTX was co-encapsulated into the micelle, followed by conjugating the angiopep-2 peptide that actively transported the antibody and PTX across the BTB to elicit an activatable immune response against GBM through redox-responsive crosslinker chain-breaking, and amplified ICB efficacy by PTX-induced ICD effect to enhance anti-GBM therapy. (**b**) Quantification of tumor-infiltrating T cell three days after two treatments with saline, free aPD-L1, free PTX, A2-AM, A2-PM, APM, and A2-APM, *n* = 3 mice. (Adapted from [147], CC BY 4.0.).

Consistent with this principle, redox-responsive polymeric micelles co-encapsulating PTX and anti-PD-L1 antibody, as shown in Figure 5, have demonstrated synchronized delivery across the blood–tumor barrier (BTB) in glioblastoma (GBM) models. In this system, Angiopep-2-mediated transcytosis and disulfide-triggered intracellular release coupled with PTX-induced ICD with preserved checkpoint inhibition within the same anatomical compartment [147]. This coordinated exposure increased cytotoxic T-lymphocyte accumulation, promoted pro-inflammatory macrophage polarization, suppressed primary and recurrent tumors, and induced durable immunological memory, illustrating how micellar PTX combinations transform chemotherapy into a spatially and temporally synchronized chemo-immunotherapeutic amplifier.

A representative example of synchronized chemo–gene immunotherapy was reported by Sun et al., who developed a TME-triggered charge-reversal polymetformin-based micellar nanosystem for co-delivery of DOX and the IL-12 plasmid (pIL-12) [151]. The cationic polymetformin core enabled simultaneous DOX encapsulation and electrostatic condensation of pIL-12, while hyaluronic acid shielding enhanced systemic stability and CD44-mediated tumor targeting. Upon hyaluronidase-triggered deshielding in the TME, charge reversal promoted cellular uptake and intracellular release. This spatial coordination aligned DOX-induced ICD with intratumoral IL-12 expression, resulting in enhanced M1 macrophage polarization, increased CD8^+^ T cell and NK cell infiltration, reduced Treg prevalence, and superior antitumor and antimetastatic efficacy compared with either modality alone. Here, micellar co-delivery did not merely improve formulation feasibility but synchronized cytotoxic and cytokine-driven immune activation within the same tumor niche.

**Figure 6:**
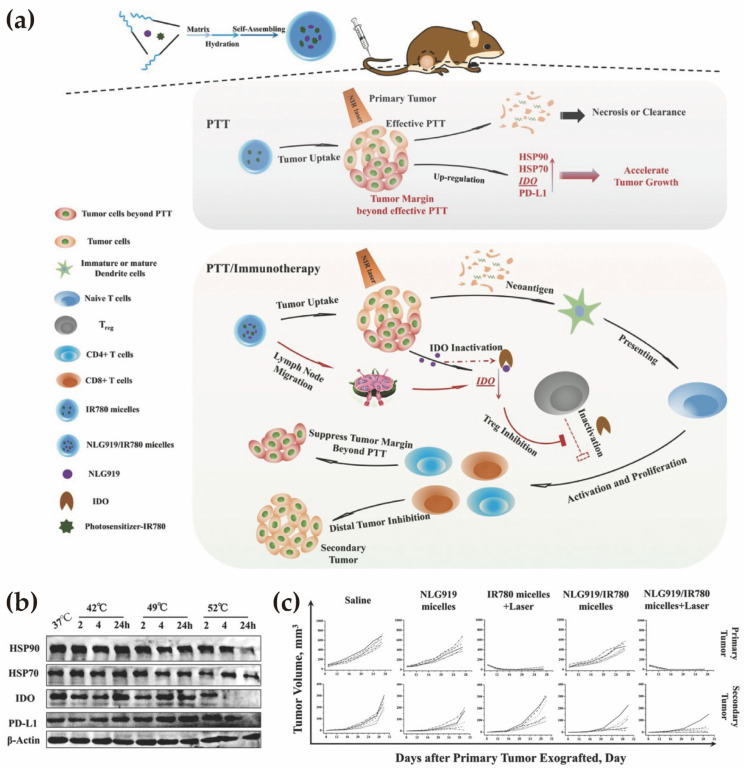
ROS-amplified photoimmunotherapy via IDO inhibition suppresses tumor margins beyond effective PTT and induces systemic antitumor immunity. (**a**) Scheme of the preparation pathway of NLG919(IODI)/IR780 coloaded micelles and the mechanism by which the NLG919/IR780-micelle-mediated PTT, combined with immunotherapy, suppressed the growth of the tumor margin beyond effective PTT and the distal (or secondary) tumor. Arrows indicate the relationships and processes described in the schematic; their meanings are specified by the accompanying labels. (**b**) Tumor volume of the mice treated with saline and with PTT (partially treated). (**c**) Tumor volume of primary tumor and secondary tumor versus time, respectively (*n* = 6). (Adapted from [196], licensed under CC BY 4.0.).

A second mechanistically distinct example was provided by Peng et al., that co-encapsulated the hydrophobic photosensitizer IR780 and the IDO inhibitor NLG919 within mPEG–*b*–PCL micelles (Figure 6a) [196]. PTT alone was shown to induce adaptive immune resistance in residual tumor margins, characterized by upregulation of IDO and PD-L1 (Figure 6b). Micellar co-delivery enabled concurrent intratumoral accumulation of IR780 and NLG919, such that therapy-induced metabolic immune escape was counteracted in situ. When combined with laser irradiation, synchronized exposure enhanced CD8^+^ T cell infiltration, reduced Treg populations, suppressed both primary and distant tumors, and promoted systemic antitumor immunity (abscopal effect) (Figure 6c). This study illustrates how micelles can align therapy-induced stress responses with checkpoint or metabolic blockade, converting local cytotoxic intervention into coordinated immune activation.

A particularly instructive example of spatially programmed exposure synchronization was reported by Wang et al., who engineered a dual pH-responsive polymeric nanosystem for sequential release of anti-PD-L1 antibody and DOX [203]. The amphiphilic polymer incorporated two ionizable segments with distinct pKa values, enabling staged disassembly under extracellular tumor acidity (pH~6.8–6.9) and subsequent endolysosomal acidification (pH~5.0–5.5). Anti-PD-L1 was preferentially released in the mildly acidic tumor interstitium to achieve checkpoint blockade at the tumor–immune interface, whereas DOX was retained until intracellular acidification triggered ICD within tumor cells. This compartmentalized release minimized premature antibody degradation while maximizing cytotoxic immunogenic signaling. Mechanistically, therapeutic efficacy was dependent on BATF3^+^ dendritic cells and was associated with enhanced cross-presentation and robust CD8^+^ T cell priming, demonstrating that micellar architectures can be programmed to align drug action sites across tumor compartments.

Beyond these representative systems, additional studies reinforce the principle of exposure synchronization. pH-responsive PLGA–poly(histidine) nanoparticles co-delivering PTX and resiquimod demonstrated coordinated cytotoxicity and innate immune activation in breast cancer models [204]. Micellar PTX formulations have been shown to preserve ICD while reducing immune cell toxicity and, when combined with anti-PD-1 therapy, enhance CD8^+^ T cell-dependent tumor control. Integrin-targeted reduction-responsive PTX micelles paired with nano-formulated STING agonists further amplified innate and adaptive immune responses in TNBC. Similarly, poly(2-oxazoline)-based micelles co-formulating idelalisib and resiquimod revealed that single-drug solubilization improved systemic handling but that pronounced tumor eradication emerged only when both immunomodulators were co-encapsulated and synchronously delivered, particularly in combination with radiotherapy [145]. Across these systems, therapeutic amplification consistently arose not from combination alone, but from micelle-mediated alignment of spatial and temporal drug exposure within the TME.

Taken together, these examples establish polymeric micelles as programmable exposure-aligning platforms rather than passive co-solubilizing carriers. By synchronizing cytotoxic stress, checkpoint inhibition, metabolic reprogramming, or cytokine signaling within shared tumor compartments, micellar co-delivery architectures transform parallel therapies into integrated immunological circuits, thereby enhancing efficacy while limiting systemic toxicity.

## 4. Discussion

Small-molecule immunomodulators have transformed modern immunotherapy by targeting immune checkpoints, metabolic pathways, cytokine signaling networks, and intracellular kinases. However, a recurring pattern across diverse mechanistic classes is that therapeutic limitations frequently arise not from insufficient target engagement, but from suboptimal exposure profiles and lack of spatial control within diseased tissues. Whether in checkpoint inhibition, metabolic reprogramming, or innate immune activation, systemic administration often fails to generate the precise temporal and tissue-restricted signaling required for effective immune modulation.

The classification presented in this review highlights that delivery constraints are not uniformly distributed but are tightly coupled to the biological mechanism of each target class. Highly lipophilic checkpoint modulators and kinase inhibitors are limited by poor aqueous solubility and excipient-dependent formulations. Innate immune agonists such as TLR and STING ligands face challenges related to charge, permeability, and systemic inflammatory toxicity. Metabolic modulators, including IDO1, arginase, and adenosine pathway inhibitors, frequently demonstrate that adequate systemic exposure does not necessarily translate into effective intratumoral pathway inhibition. Collectively, these examples underscore that pharmacokinetics and spatial drug distribution are often the dominant determinants of immunotherapeutic outcome.

Within this context, polymeric micelles should not be viewed merely as solubilizing excipients but as programmable delivery architectures capable of modulating exposure, release kinetics, and tissue distribution. Solubilization-driven micelles expand the chemical space of immunomodulatory agents amenable to systemic administration, particularly for hydrophobic molecules. Stimuli-responsive micelles translate tumor-specific biochemical cues—such as acidic pH, redox gradients, and enzymatic activity—into controlled drug release, effectively coupling microenvironmental signals to immune reprogramming. Spatially targeted micelles enable selective delivery to tumor cells, immune cell subsets, or inflamed tissues, mitigating systemic immune perturbation. Finally, co-delivery systems facilitate pharmacokinetic synchronization, aligning cytotoxic, metabolic, and immune-modulating mechanisms within a shared spatial and temporal window (Table 1).

Importantly, the distinction between direct and indirect immunomodulation emerges as a conceptual framework for micelle-enabled strategies. Direct immunomodulators, such as checkpoint or metabolic inhibitors, require controlled exposure to avoid systemic immune dysregulation. In contrast, cytotoxic agents capable of inducing ICD exert indirect immunomodulatory effects that can be amplified by micellar delivery through enhanced intracellular uptake and spatial confinement. By integrating both direct and indirect mechanisms within rationally engineered platforms, polymeric micelles offer a route to transform local tumor stress into systemic immune activation while maintaining safety.

Despite promising preclinical advances, several translational challenges remain. Manufacturing reproducibility, scalability of complex micellar architectures, stability during storage and circulation, and regulatory classification of multifunctional systems represent nontrivial hurdles. Moreover, clinical trial design must account for exposure synchronization and immune dynamics rather than relying solely on traditional pharmacokinetic endpoints. Addressing these issues will be essential to translate micelle-based immunomodulatory strategies from experimental platforms to standardized clinical modalities.

## 5. Perspectives and Future Directions

The preceding discussion establishes that polymeric micelles possess the architectural versatility to address delivery constraints across mechanistically diverse small-molecule immunomodulators. However, translating these preclinical advances into clinically viable immunotherapeutic strategies requires confronting several interconnected challenges spanning manufacturing, regulation, long-term safety, and clinical trial design. This section examines key translational barriers and identifies opportunities that may guide the next phase of micelle-enabled immunotherapy development.

### 5.1. Lessons from Clinical Experience: Successes, Setbacks, and Unresolved Questions

The clinical trajectory of polymeric micelle formulations offers both validation and cautionary lessons for the immunomodulatory applications discussed in this review. Genexol-PM, a PEG–*b*–PDLLA micellar formulation of PTX, achieved regulatory approval in South Korea in 2007 and has since been evaluated in multiple phase II and III trials across breast cancer, non-small-cell lung cancer, ovarian cancer, and pancreatic cancer [20]. Its approval demonstrated that Cremophor-free micellar delivery could preserve cytotoxic efficacy while substantially reducing hypersensitivity reactions and neutropenia associated with conventional surfactant-based formulations [21]. However, Genexol-PM did not demonstrate superior overall survival compared with conventional PTX in randomized trials, indicating that improved tolerability does not automatically translate into superior efficacy.

The phase III experience of NK105, a PEG–*b*–poly(aspartate) micellar PTX, further illustrates this challenge. Despite favorable preclinical pharmacokinetics and reduced peripheral neuropathy (1.4% vs. 7.5% grade 3/4), NK105 failed to meet its primary endpoint of non-inferiority in progression-free survival compared with conventional PTX in metastatic breast cancer (n = 436) [22]. This outcome highlighted a fundamental disconnect between preclinical tumor accumulation and clinical efficacy, demonstrating that improved pharmacokinetics alone are insufficient when the therapeutic payload does not inherently require spatially restricted exposure to exert its pharmacological effect. This lesson carries significance for immunomodulators, for which therapeutic efficacy is governed by spatial and temporal drug distribution within the TME rather than by systemic exposure alone.

NC-6004 (Nanoplatin), a cisplatin-incorporating PEG–*b*–poly(glutamic acid) micelle, has advanced into phase III evaluation for pancreatic and head-and-neck cancers, demonstrating reduced nephrotoxicity and neurotoxicity compared with free cisplatin [22]. SP1049C, a Pluronic-based micellar DOX formulation with P-glycoprotein inhibitory properties, has also progressed to advanced clinical evaluation for gastric and esophageal adenocarcinoma [205]. Taken together, these clinical experiences confirm that micellar delivery can reproducibly improve safety profiles. Whether this safety advantage can be exploited to enable immunomodulatory dosing regimens that would otherwise be dose-limited by systemic toxicity remains a compelling but as yet unanswered question.

Notably, none of the polymeric micelle formulations currently in clinical trials were specifically designed for immunomodulatory applications. Future clinical programs should consider incorporating immunological endpoints such as intratumoral immune cell infiltration, peripheral immune biomarkers, and cytokine profiling alongside conventional pharmacokinetic and tumor response criteria to capture the unique therapeutic dimensions of micelle-enabled immunotherapy.

### 5.2. Manufacturing Scalability and Process Reproducibility

The translational viability of polymeric micelle systems depends on the ability to manufacture them at clinical and commercial scales with acceptable batch-to-batch consistency. Conventional preparation methods, such as thin-film hydration, solvent evaporation, and dialysis, while effective at the laboratory scale, introduce variability in critical quality attributes (CQAs), including particle size distribution, drug loading, and encapsulation efficiency, when scaled beyond milligram quantities [24]. For multifunctional micelles incorporating stimuli-responsive linkages, targeting ligands, or co-encapsulated agents as discussed in Section 3.2, Section 3.3 and Section 3.4 of this review, these challenges are compounded by the need to control multiple structural parameters simultaneously.

Microfluidic mixing technologies have emerged as a promising approach to address these limitations, offering precise control over mixing kinetics, supersaturation, and self-assembly conditions within confined geometries [206]. Continuous-flow microfluidic platforms enable real-time adjustment of critical process parameters such as flow rate ratios, total flow rate, and solvent composition, thereby reducing batch variability and facilitating Quality by Design (QbD) approaches to micellar nanomedicine manufacturing [207]. However, throughput limitations of single-channel microfluidic devices remain a barrier to commercial-scale production, and parallelization or numbering-up strategies require further validation for complex micellar architectures.

Equally important is the development of robust analytical methods for in-process and release testing. CQAs for immunomodulatory micelles extend beyond conventional physicochemical characterization (size, zeta potential, drug content) to include assessment of stimuli-responsive behavior, ligand density and accessibility, drug release kinetics under physiologically relevant conditions, and stability of co-encapsulated agents with distinct physicochemical properties. Establishing validated analytical frameworks for these complex systems will be essential for regulatory acceptance and clinical translation.

### 5.3. Regulatory Considerations for Multifunctional Micellar Systems

The regulatory framework governing polymeric micelle-based nanomedicines continues to evolve, and existing classification systems do not always accommodate the multifunctional and combination-product characteristics of advanced micellar systems. Regulatory agencies, including the FDA and the European Medicines Agency, have increasingly adopted case-by-case, risk-based evaluation approaches for nanomedicines, but specific guidance documents for polymeric micelle products remain limited compared with those available for liposomal formulations [208].

A key regulatory challenge arises when micellar systems function as combination products, for example, co-delivery platforms encapsulating two pharmacologically active agents with distinct mechanisms of action, or targeted micelles bearing surface ligands that serve both delivery and pharmacological functions. Such systems may fall under multiple regulatory pathways (drug, device, biologic), complicating the approval process. Furthermore, the dynamic equilibrium nature of polymeric micelles in which unimer–micelle exchange occurs continuously raises questions about how to define and characterize the “active pharmaceutical ingredient” in regulatory terms [24].

To facilitate regulatory acceptance, developers of immunomodulatory micelle systems should pursue early engagement with regulatory agencies through pre-investigational new drug meetings, adopt QbD principles from the outset of development, and proactively define specifications that encompass both conventional CQAs and immunological activity parameters. In addition, the co-development of companion diagnostics such as biomarkers for patient stratification based on TME characteristics may streamline both regulatory review and clinical development by enabling biomarker-guided enrichment strategies in clinical trials.

### 5.4. Long-Term Safety, Immunogenicity, and Pharmacokinetic Considerations

While polymeric micelles generally demonstrate favorable acute safety profiles in clinical settings, long-term safety considerations become particularly relevant for immunomodulatory applications that may require chronic or repeated dosing. Three interrelated concerns warrant systematic investigation: polymer accumulation, anti-PEG immunogenicity, and immune system modulation by carrier materials.

The accelerated blood clearance (ABC) phenomenon, whereby repeated administration of PEGylated nanoparticles elicits anti-PEG IgM antibodies that promote rapid hepatosplenic clearance of subsequent doses, has been well-documented for PEGylated liposomes [209]. Notably, polymeric micelles appear to exhibit a lower propensity for ABC induction compared with liposomes, potentially due to differences in complement activation and B-cell stimulation related to particle number, surface curvature, and PEG conformation [210]. Nevertheless, the prevalence of pre-existing anti-PEG antibodies in the general population, estimated at 25–70% depending on the assay and population studied, raises concerns about variable pharmacokinetics and diminished efficacy in unselected patient populations [211]. Alternative hydrophilic polymers, including poly(2-oxazoline)s, polysarcosine, and zwitterionic polymers, are under investigation as antifouling coatings that may circumvent anti-PEG immunity while preserving long circulation times.

For immunomodulatory applications specifically, a further consideration is whether the carrier itself modulates immune responses in ways that interact with the intended pharmacological effect. Certain block copolymers, notably Pluronics, have demonstrated intrinsic immunomodulatory activity through effects on P-glycoprotein, mitochondrial function, and NF-κB signaling [205,212]. While such properties may be therapeutically advantageous in specific contexts (as exemplified by SP1049C), they complicate the attribution of observed immune effects to the encapsulated payload versus the carrier. Systematic evaluation of carrier immunogenicity and immunomodulatory potential should therefore be incorporated into the preclinical development of micelle-based immunotherapeutics.

### 5.5. Polymeric Micelles in the Context of Competing Delivery Platforms

Polymeric micelles represent one of several nanoscale delivery platforms investigated for immunomodulatory applications. An objective assessment of their translational potential requires comparison with competing technologies, including liposomes, LNPs, antibody–drug conjugates (ADCs), and polymersomes, each of which offers distinct advantages and limitations for immune-directed drug delivery (Table 2).

Liposomes represent the most clinically established nanoparticle platform, with over 20 approved products including Doxil^®^ (PEGylated liposomal DOX) and, more recently, the mRNA-LNP COVID-19 vaccines [213]. Their well-characterized manufacturing processes, regulatory precedent, and demonstrated clinical safety provide significant translational advantages. However, conventional liposomes offer limited options for stimuli-responsive release and typically require cryogenic storage, which constrains deployment in resource-limited settings. LNPs have achieved transformative clinical success as delivery vehicles for nucleic acid therapeutics, particularly mRNA vaccines and siRNA drugs (patisiran) [214]. Their ionizable lipid components enable efficient endosomal escape and cytoplasmic delivery, properties that are less critical for small-molecule immunomodulators that typically act on extracellular targets or require simple intracellular release. LNPs also exhibit inherent immunostimulatory properties through activation of pattern recognition receptors, which may be advantageous for vaccine applications but problematic for immunosuppressive or precisely targeted immunomodulatory strategies. ADCs, with 14 FDA-approved products as of 2025, offer target specificity through antibody-mediated recognition but are inherently limited to cell-surface antigens and predominantly deliver cytotoxic payloads through receptor-mediated endocytosis [215]. Their DAR (typically 2–8) constrains payload capacity, and they are not readily adapted for co-delivery of agents with distinct pharmacological mechanisms, which is a key advantage of micellar co-delivery systems. Polymersomes, self-assembled vesicles formed from amphiphilic block copolymers, share chemical tunability with micelles but offer a bilayer membrane that can encapsulate both hydrophilic (aqueous lumen) and hydrophobic (membrane) cargo [216]. Their enhanced membrane stability compared with liposomes is advantageous for sustained circulation, but higher structural rigidity may limit stimuli-responsive disassembly and drug release kinetics.

Within this competitive landscape, polymeric micelles occupy a distinctive niche characterized by synthetic modularity, high drug LC for hydrophobic small molecules, dynamic self-assembly enabling stimuli-responsive behavior, and the capacity for pharmacokinetic synchronization of co-encapsulated agents. These properties are particularly well-aligned with the delivery requirements of small-molecule immunomodulators as discussed throughout this review. However, the clinical maturity of competing platforms such as liposomes and LNPs means that polymeric micelles must demonstrate clear therapeutic advantages beyond formulation convenience to justify the development investment required for clinical translation.

Beyond oncology-focused applications, the co-delivery capabilities of polymeric micelles may extend to vaccine development. Micellar platforms can co-encapsulate subunit antigens with molecular adjuvants such as TLR agonists (e.g., resiquimod, MPLA) or STING agonists within a single nanocarrier, enabling synchronized delivery to antigen-presenting cells and potentially enhancing antigen-specific immune priming [14,72,74]. As discussed in Section 3.3, polymeric nanoformulations have already demonstrated the ability to promote dendritic cell activation and antigen presentation via MHC I pathways while restricting innate immune activation to lymphoid compartments. These attributes position polymeric micelles as candidate adjuvant platforms for both prophylactic and therapeutic vaccines, a direction that warrants further investigation to align with the broader scope of immunomodulatory delivery.

## 6. Conclusions

Small-molecule immunomodulators represent a mechanistically diverse yet pharmacologically constrained class of therapeutics whose efficacy is frequently limited by physicochemical properties and insufficient spatial control. Across adaptive, innate, metabolic, and kinase-directed pathways, delivery barriers—rather than target validity—often dictate clinical performance.

Polymeric micelles provide a broadly adaptable platform to overcome these constraints. Through enhanced solubilization, stimuli-responsive release, spatial targeting, and co-delivery capabilities, micellar systems enable more precise orchestration of immune modulation in vivo. By bridging immunopharmacology with formulation science, polymeric micelles extend beyond passive drug carriers and function as active regulators of when, where, and how immunomodulatory signals are perceived.

As immunotherapy continues to evolve toward combination-based and microenvironment-aware strategies, rationally engineered micellar platforms hold promise for achieving tumor-restricted immune activation while minimizing systemic toxicity. Future advances will depend on integrating materials design with mechanistic immunology, developing scalable manufacturing approaches, and designing clinical trials that capture the unique spatiotemporal advantages of micelle-enabled delivery. Through such efforts, polymeric micelles may emerge as a generally translatable strategy for enhancing the safety and efficacy of next-generation small-molecule immunomodulators.

## Figures and Tables

**Table 1:** Micelle design strategies and immunological consequences.

Design Strategy	Polymer/Platform	Cargo	Physicochemical Properties	Key Trigger/Mechanism	Immunological Outcome	Reference
Solubilization-driven	PEG–*b*–PBLG	Tranilast	~90–95 nm; LC~10 wt%; PDI~0.12; t_½_ ≈ 21 h	Hydrophobic core stabilization; PK extension	CAF reprogramming; ↓ tumor stiffness; ↑ CD8^+^ T cell infiltration	[144]
High-capacity solubilization	Poly(2-oxazoline) micelles	Resiquimod + Idelalisib	27–34 nm; LC > 40 wt%; LE > 90 wt%	Hydrophobic matching; high LC	Enables systemic dosing; foundation for combination therapy	[145]
Dual pH-responsive	ML-HGD/PL-HGD (mPEG–*b*–P(L-lysine) shielding)	DOX + imiquimod	~60–65 nm; staged extracellular/endolysosomal release	Protonation; PEG deshielding; hydrazone cleavage	ICD + TLR activation; M1 polarization; ↑ CD8^+^ infiltration	[146]
Redox-responsive	Angiopep-2–PEG–PLL micelles	Paclitaxel + anti–PD-L1	58.8 nm; LC PTX 47.8 wt%; LC IgG 22 wt%	GSH-triggered disulfide cleavage; BTB transcytosis	↑ CTL infiltration; pro-inflammatory macrophage shift; immune memory	[147]
ROS-amplifying	RIMNA micelles	ICG + NLG919	~160 nm; ζ ≈ −22 mV	Photothermal/ROS amplification; metabolic blockade	PD-1 synergy; abscopal effect; complete tumor regression	[148]
Enzyme-responsive	PEG–*b*–poly(Trp) micelles	NLG919	150–190 nm; IDO-triggered swelling	IDO-mediated oxidation; core hydrophilization	↓ Kyn/Trp ratio; ↑ CD8^+^ T cell infiltration	[149]
Spatial targeting	α5β1 integrin-targeted micelles	PTX	LC 23.1 wt%; redox-crosslinked core	Integrin targeting + intracellular reduction	Enhanced ICD; ↓ Tregs; TNBC immune reprogramming	[150]
Co-delivery synchronization	Polymetformin-based charge-reversal micelles	DOX + pIL-12	Tumor-triggered charge reversal system	Hyaluronidase activation; staged intracellular release	Chemo–gene immune amplification; ↑ CD8^+^/NK cells; ↓ Tregs	[151]

(↓) Decrease/Downregulation; (↑) Increase/Upregulation.

**Table 2:** Comparative assessment of nanoscale delivery platforms for small-molecule immunomodulators.

Feature	Polymeric Micelles	Liposomes	LNPs	Antibody–Drug Conjugates	Polymersomes
Typical size (nm)	10–100	80–200	50–150	5–10	50–300
Drug loading capacity	Moderate–High (5–45 wt%)	Low–Moderate (1–10 wt%)	Variable (nucleic acids primarily)	Low (drug-to-antibody ratio 2–8)	Moderate (hydrophilic + hydrophobic)
Small-molecule compatibility	Excellent (hydrophobic core)	Good (bilayer + lumen)	Limited (optimized for nucleic acids)	Good (linker-conjugated)	Good (membrane + lumen)
Stimuli-responsive release	Highly tunable (pH, redox, ROS, enzyme)	Limited (pH-sensitive variants)	Limited (ionizable lipid pH)	Moderate (cleavable linkers)	Moderate (membrane engineering)
Co-delivery capability	Excellent (PK synchronization)	Good (lumen + bilayer)	Good (nucleic acid combinations)	Poor (single payload per Ab)	Good (dual compartment)
Targeting strategy	Passive (EPR ^(1)^) + active ligand decoration	Passive (EPR) + antibody/peptide	Passive (liver tropism) + emerging targeting	Active (antibody-mediated)	Passive + active (surface functionalization)
Manufacturing scalability	Moderate (microfluidics emerging)	Established (ethanol injection, thin-film)	Established (microfluidic mixing)	Complex (bioconjugation required)	Moderate (similar to micelles)
Regulatory precedent	Limited (Genexol-PM, Nanoxel)	Extensive (Doxil, Onivyde, 20+ products)	Extensive (Comirnaty, Spikevax, Onpattro)	Extensive (14 FDA-approved)	Minimal (preclinical)
Storage stability	Good (lyophilizable)	Variable (often 2–8 °C)	Limited (often −20 °C to −80 °C)	Good (2–8 °C)	Good (lyophilizable)
Immunomodulatory applications	Emerging (ICD, checkpoint, metabolic co-delivery)	Established (adjuvants, vaccines)	Transformative (mRNA ^(2)^ vaccines, CAR-T)	Emerging (immuno-conjugates)	Early (preclinical)
Key limitations	CMC dilution, ABC risk, clinical maturity gap	Low loading, cold chain, limited stimuli-response	Liver tropism, cold chain, innate immune stimulation	Low payload, surface antigen required, cost	Slow release, manufacturing complexity

^(1)^ Enhanced permeability and retention; ^(2)^ Messenger RNA.

## Data Availability

No new data were created or analyzed in this study.

## Abbreviations

A2AR	A2A adenosine receptors
ABC	accelerated blood clearance
ADC	antibody–drug conjugate
AhR	aryl hydrocarbon receptor
ALK5	activin receptor-like kinase 5
ATP	adenosine triphosphate
*b*	block
BTB	blood–tumor barrier
BTK	Bruton’s tyrosine kinase
CCL	CC motif chemokine ligand
CDN	cyclic dinucleotide
CMC	critical micelle concentration
CQA	critical quality attribute
CXCL	C-X-C motif chemokine ligand
DAR	drug-to-antibody ratio
DOX	doxorubicin
EPR	enhanced permeability and retention
FDA	U.S. Food and Drug Administration
GBM	glioblastoma
GCN2	general control nonderepressible 2
GSH	glutathione
HMGB1	high mobility group box 1
ICD	immunogenic cell death
IDO1	indoleamine 2,3-dioxygenase 1
IFN	interferon
Kyn	kynurenine
LC	loading capacity
LE	loading efficiencies
LNP	lipid nanoparticle
mPEG	methoxy poly(ethylene glycol)
MPLA	monophosphoryl lipid A
mRNA	messenger RNA
NK cell	natural killer cell
PBLG	poly(benzyl-L-glutamate)
PCL	poly(ε-caprolactone)
PD-1	programmed cell death protein 1
PD-L1	programmed death-ligand 1
PDHA	poly((1,4-butanediol) diacrylate-β-5-hydroxyamylamine)
PDI	polydispersity index
PEG	poly(ethylene glycol)
PI3K-δ	Phosphoinositide 3-kinase δ
PLGA	poly(lactic-co-glycolic acid)
POx	poly(2-oxazoline)-based triblock copolymers
PTT	photothermal therapy
PTX	paclitaxel
QbD	Quality by Design
RORγt	retinoic acid receptor-related orphan receptor gamma t
ROS	reactive oxygen species
SHIP1	SH2-containing inositol 5′-phosphatase 1
STING	stimulator of interferon genes
t½	half-life
TGF-β	transforming growth factor-β
TLR	toll-like receptor
TME	tumor microenvironment
TNBC	triple-negative breast cancer
tranilast/m	tranilast-loaded micelles
Treg	regulatory T cell
Trp	tryptophan
